# Quasi-Distributed Partial Discharge Monitoring System with Remote Demodulation Based on DFB-FL/Interferometer Hybrid Sensing

**DOI:** 10.3390/nano16150937

**Published:** 2026-07-29

**Authors:** Yuelan Lu, Qibing Shao, Qun Yu, Hongliang Zhang, Xiaolong Zhang, Huagang Zhan, Weichao Zhang

**Affiliations:** 1 School of Low-Altitude Technology and Electronic Information, Guangdong University of Science and Technology, Dongguan 523668, China; luyuelan1209@163.com; 2 Zhongtian Technology Submarine Cable Co., Ltd., Nantong 226010, China; cyrus.shao@zttgroup.com (Q.S.);; 3 Key Laboratory of Engineering Dielectrics and Its Application, Ministry of Education, Harbin University of Science and Technology, Harbin 150001, China; 2210302057@stu.hrbust.edu.cn

**Keywords:** polymer insulation, partial discharge, acoustic signal, DFB-FL, interferometer

## Abstract

In the field of long-distance partial discharge (PD) detection for submarine cables, there is an urgent need for a remote demodulation distributed detection technology deployable at multiple critical locations to overcome the limitations of single-point measurement. Factory joints of high-voltage submarine cables are high-risk components for PD, and long-distance fiber optic acoustic sensing technology holds the greatest potential for online monitoring. However, due to the viscoelasticity of the joint’s polymer insulation structure, sound propagation distance is severely limited, and non-multi-point measurement cannot achieve effective coverage of the measurement area. This paper proposes a quasi-distributed remote demodulation sensing system, in which both the fiber optic interferometer and the distributed feedback fiber laser (DFB-FL) serve as sensors, enabling highly sensitive quasi-distributed PD detection. The DFB-FL itself is highly sensitive to strain, and the multiple fiber coils formed by the interferometer arms are also highly sensitive to strain. Both can be modulated by the micro-strain induced by the acoustic field generated from PD in the polymer solid, producing phase shifts of the optical waves, which are then intrinsically demodulated by the interferometer system to extract the vibration signals caused by the discharge. Theoretical analysis shows that the sensitivity increases with the length of the unbalanced arm, with the upper limit constrained by laser coherence and optical attenuation; for the PD frequency band, the optimal unbalanced length is below 200 m—this design rule is applicable to on-chip interferometric sensors. The interferometer coils employ bend-insensitive fibers to suppress optical loss and improve fringe visibility. Meanwhile, three fiber coil configuration schemes are constructed to enhance the detection sensitivity to acoustic signals. Simulation results generate frequency response contour maps based on Young’s modulus, indicating that the solid-wound coil achieves the highest amplitude and the broadest bandwidth, with an optimal response frequency of approximately 50 kHz. Experimental results demonstrate that among the three structure types, the solid-wound coil also achieves the largest response ratio. Finally, in tests performed on a 220 kV submarine cable intermediate joint (with the system installed inside the metallic sheath), the minimum detectable discharge level in the DFB-FL region reached 6.75 pC, while that for the fiber coil reached 12.66 pC; when installed outside the metallic sheath, the minimum detectable discharge levels were 53.2 pC for the grating region and 89.6 pC for the fiber coil.

## 1. Introduction

Partial discharge is generally accompanied by multiple physical phenomena, including electrical pulses, luminescence, temperature rise, variation of gas components and mechanical vibration. The initiation and evolution of these characteristic signals are commonly adopted to identify the existence of partial discharge and quantify its intensity variation [1,2]. Compared with gaseous and liquid insulating materials, the polymer-based main insulation of power cables features low light transmittance, immobility, high heat capacity and low thermal conductivity. Such intrinsic properties make optical detection, gas-phase diagnostic methods and temperature monitoring incapable of achieving reliable real-time partial discharge identification. The fiber optic vibration (ultrasonic) monitoring approach for partial discharge presents prominent engineering applicability, owing to its miniature size, zero introduction of extra conductive components and outstanding anti-electromagnetic-interference performance [3,4,5,6,7,8,9,10,11,12,13,14].

Nevertheless, the core bottleneck of vibration-based monitoring lies in the viscoelastic behavior of polymers under vibration excitation. Relative displacement inevitably occurs between distinct molecular chain segments, crystalline regions and amorphous domains, as well as between the polymer matrix and inorganic fillers. Weak interfacial interactions between these heterogeneous phases repeatedly break and recover during the relative motion, which converts the mechanical energy carried by the incident vibration wave into internal energy. This energy attenuation mechanism results in the poor propagation capacity of vibration signals, which cannot reach positions far away from the discharge source [14]. Point-type sensors represented by DFB-FL have been verified to possess ultra-high measurement sensitivity. Restricted by the dielectric characteristics of polymer materials, the effective monitoring range of a single point sensor is extremely limited, and the expansion of sensing coverage is therefore an urgent requirement [8,14].

Sensing schemes capable of covering large-scale monitoring areas are mainly divided into interferometer-type sensors and distributed optical fiber sensing technologies [15,16,17,18]. In 2022, the research team led by Xu Yang applied distributed sensing technology to partial discharge fault diagnosis for 220 kV cable joints, and successfully captured partial discharge signals with an apparent discharge magnitude of 124 pC [19]. In 2023, Ma Guoming et al. wound single-mode optical fibers on the outer semi-conductive layer of cable joints. Coherent optical time-domain reflectometry (COTDR) was employed to monitor partial discharge induced by conductor protrusions and air gaps. Via Fourier integration and differential calculation, the acoustic waveform characteristics of partial discharge corresponding to the two typical defect types were extracted with high accuracy [20]. In 2024, researchers from Hebei University established a φ-OTDR fiber optic ultrasonic sensing system equipped with a polarization extinction unit. The random variance of Rayleigh scattered light was selected as the characteristic indicator for weak signal detection. Optical fibers were arranged around cable joints, and artificial defect samples were fabricated. The system acquired ultrasonic signals corresponding to partial discharge with an apparent discharge magnitude of 3650 pC, and realized a localization accuracy of 1.9 m [21]. Distributed optical fiber sensing technology exhibits distinct merits and inherent defects. Its advantages lie in the ultra-large measurable coverage and simple deployment procedure, as the sensing unit only consists of optical fiber. Its shortcomings are reflected in the practical engineering application scenarios with long-distance and large-scale layouts: active optical amplifiers are required for long-distance transmission and high-frequency pulse light sources are indispensable for high-frequency signal acquisition, which results in high deployment cost and limited adaptability for the detection of minor partial discharge at key equipment positions. Interferometer-type sensors contain three mainstream configurations: the Mach–Zehnder fiber interferometer, Michelson interferometer and Sagnac interferometer. Quasi-distributed measurement with a wide monitoring range can be realized by optimizing the layout scheme of the measurement arm. In 2021, Hao Yanpeng deployed the measurement arm of a Mach–Zehnder fiber interferometer inside the terminal of a 10 kV XLPE (cross-linked polyethylene) cable to conduct partial discharge monitoring. Test results demonstrate that the minimum detectable apparent discharge magnitude of the proposed fiber optic sensing system reaches 196 pC, and the detected inception voltage of partial discharge is 27.5% lower than the result obtained by the current transformer [22]. Although interferometer-based sensors feature low manufacturing cost and satisfactory detection performance for key monitoring locations, their sensing sensitivity and signal-to-noise ratio are severely constrained by the output linewidth of the laser and thermal noise inside the test platform [23,24,25]. When the light source is placed far away from the monitoring region, frequency dispersion and laser linewidth broadening will occur during optical transmission, which will degrade the overall detection sensitivity.

The factory joint of high-voltage submarine cables is one of the key application scenarios for partial discharge monitoring of polymer insulation. Although its length is only approximately 600–800 mm, it is the position with the most manual participation, the highest technical difficulty and the highest failure probability during the entire production process of submarine cables, making partial discharge monitoring for such joints of great practical engineering significance. Long distances between measurement points and observation points, narrow installation spaces for sensors, weak acoustic signals generated by partial discharge, and the complex transmission environment of signal propagation paths are the major engineering challenges faced by acoustic partial discharge monitoring of submarine cables. This paper takes the factory joints of high-voltage submarine cables as the research object and develops a high-sensitivity multi-point distributed sensing test system. The proposed scheme deeply integrates two high-sensitivity detection methods, namely the phase demodulation-based DFB-FL point measurement method and the optical fiber interferometer measurement method. The unbalanced optical fiber interferometer is applied to demodulate the wavelength variation of the DFB-FL nearby, which avoids the anti-interference deficiency of DFB-FL phase demodulation systems during long-distance transmission. Meanwhile, the DFB-FL provides a narrow-linewidth light source for the interferometer at the proximal end, which eliminates various nonlinear effects induced by the long-distance transmission of high-power narrowband lasers, thereby establishing a high-sensitivity multi-point distributed sensing test system suitable for partial discharge monitoring of submarine cable intermediate joints.

## 2. Principle of the DFB-FL/Interferometer Composite Partial Discharge Test System

### 2.1. Basic Structure of the DFB-FL/Interferometer Composite Partial Discharge Test System

**Figure 1:**
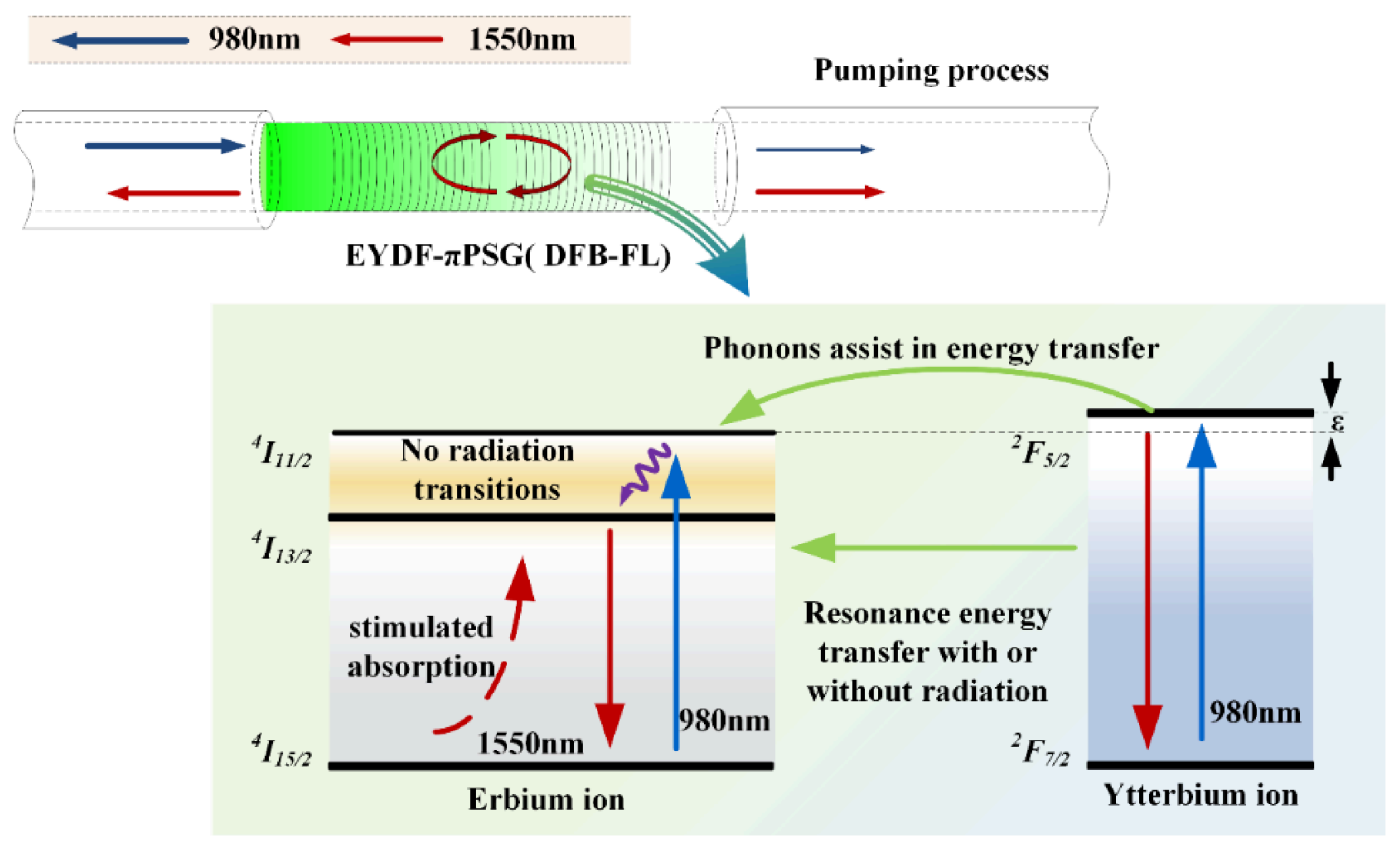
Basic structure of the distributed feedback fiber laser.

This paper proposes a DFB-FL/interferometer composite integrated sensing and demodulation test system based on collaborative sensing between the DFB-FL and interferometer. As shown in Figure 1, the basic structure of the distributed feedback fiber laser is a phase-shifted grating with a phase shift of π written on an erbium–ytterbium co-doped fiber. When a 980 nm pump laser irradiates the doped fiber, the gain medium undergoes complex pumping processes including stimulated excitation, non-radiative relaxation, radiative and non-radiative energy transfer between erbium and ytterbium ions, and phonon-assisted energy transfer, producing a broadband laser at a 1550 nm wavelength. Under the resonant enhancement effect of the equivalent resonant cavity formed by the phase-shifted grating, a narrowband laser satisfying the resonance condition is emitted from both sides of the grating. Compared with the DFB-FL based on erbium-doped fiber, the DFB-FL constructed from erbium–ytterbium co-doped fiber has higher energy conversion efficiency and a more stable output laser. The magnitude of the output laser from the left and right sides of the laser is determined by the phase-shift point position. When the phase-shift point is located at the center of the grating region, the output light intensity from the left and right sides is equal.

**Figure 2:**
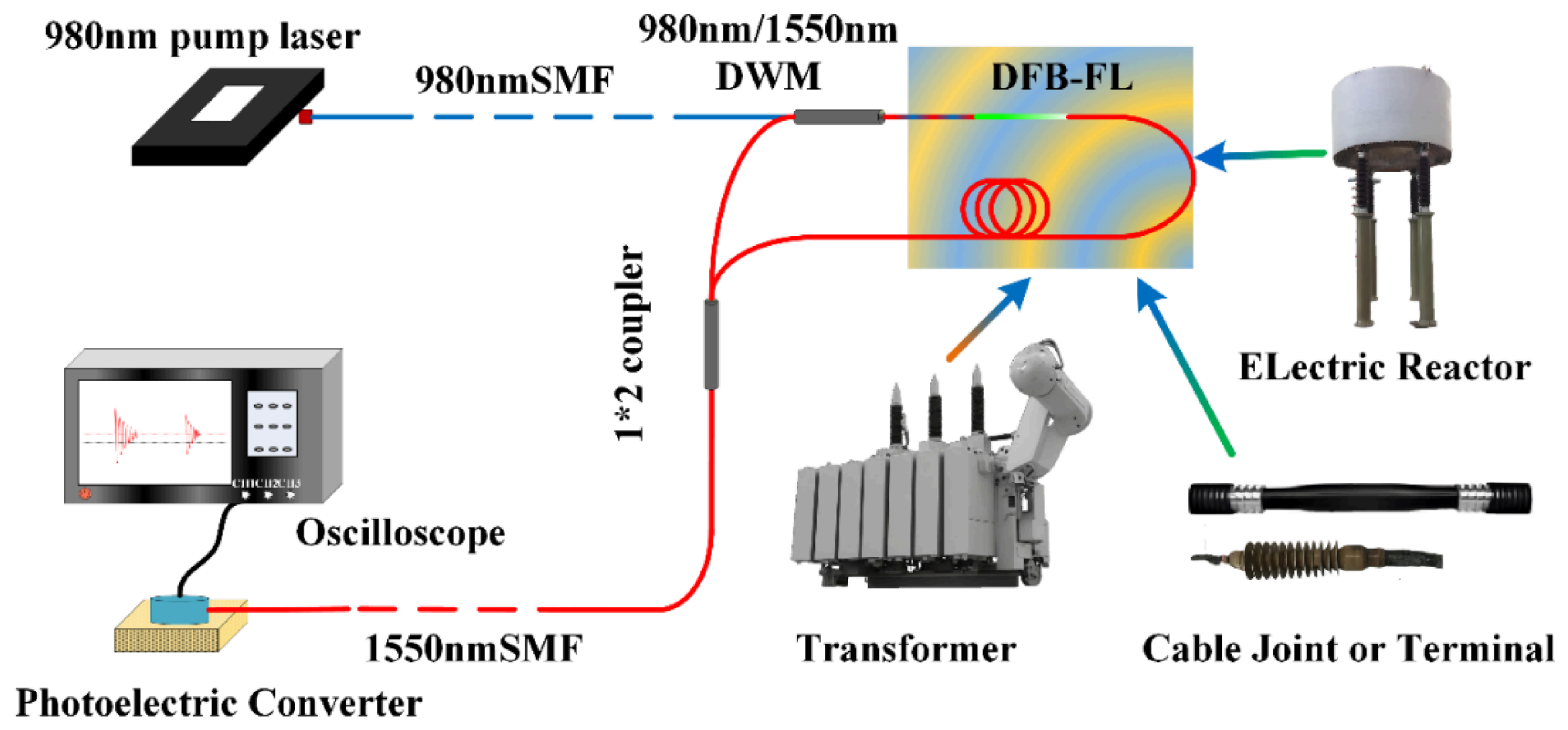
Schematic diagram of remote demodulation structure for DFB-FL/interferometer.

This paper proposes the basic sensing system structure based on the DFB-FL/interferometer shown in Figure 2. The fiber optic interferometer in this system is a Mach–Zehnder interferometer, whose basic principle is to combine two beams of light with identical wavelength but different phase through different rectangular optical paths and then into a coupler to form an interferometric beam. The variation in coupled laser intensity reflects the magnitude of the phase difference, which for light from the same source is usually provided by unequal interferometer arm lengths. For a DFB-FL with the phase-shift point at the center of the grating region, the output lasers from the left and right sides entering the coupler through different transmission paths naturally form a Mach–Zehnder interferometric structure. In this case, the interferometer not only measures external vibration through the interferometer arms, but also demodulates wavelength fluctuations of the DFB-FL caused by vibration. Based on the above principles, the system consists of a 980 nm or 1480 nm pump source, a wavelength division multiplexer, a DFB-FL, fiber loops wound from single-mode fiber for the interferometer arms, a 2 × 2 coupler, a 1550 nm fiber isolator, a photoelectric converter, a filter circuit, and a host computer. The choice of pump wavelength depends primarily on the propagation distance of the pump light: a 1480 nm pump laser is selected for longer distances because of its lower transmission attenuation, while a 980 nm laser is chosen for shorter distances because of its higher pumping efficiency. The basic topological structure of the test system is shown in Figure 1. The basic structure of the distributed feedback fiber laser is a phase-shifted grating with a phase shift of π written on an erbium–ytterbium co-doped fiber. When the 980 nm pump laser irradiates the doped fiber, the gain medium undergoes complex pumping processes including stimulated excitation, non-radiative relaxation, radiative and non-radiative energy transfer between erbium and ytterbium ions, and phonon-assisted energy transfer, producing a broadband laser at a 1550 nm wavelength. Under the resonant enhancement effect of the equivalent resonant cavity formed by the phase-shifted grating, a narrowband laser satisfying the resonance condition is emitted from both sides of the grating.

The basic principle of the fiber optic interferometer is to combine two beams of light with identical wavelength but different phase through a coupler into a single beam. The variation in coupled laser intensity reflects the magnitude of the phase difference, which for light from the same source is usually provided by unequal interferometer arm lengths. For a DFB-FL with the phase-shift point at the center of the grating region, the output lasers from the left and right sides entering the coupler through different transmission paths naturally form an interferometric structure. In this case, the interferometer not only measures external vibration through the interferometer arms, but also demodulates wavelength fluctuations of the DFB-FL caused by vibration. Based on the above principles, this paper proposes the basic sensing system structure based on a DFB-FL and interferometer shown in Figure 2. It consists of a 980 nm pump source, a 980/1550 nm wavelength division multiplexer, a DFB-FL with the phase-shift point at the center of the grating region, a 1 × 2 coupler, a 1550 nm optical isolator, a photoelectric converter, a filter circuit, and a host computer (usually an oscilloscope). The sensing elements of this system are the DFB-FL and one or both interferometer arms (sensing arms), which can be placed at cable joints, terminals, dry-type reactors, and other locations relying on polymer insulation, or at transformers, wet-type reactors, and other locations relying on liquid insulation. It is worth noting that the phase in the optical fiber is far more sensitive to vibration signals than the intensity, so placing the demodulation structure at the remote end can effectively avoid interference from phase signals on the long transmission line to the test results.

### 2.2. Sensing Principle of the DFB-FL/Interferometer Composite Partial Discharge Test System

According to the basic principle of the interferometer, the variation in interferometric light intensity detected by the photodetector is given by Equation (1).

(1) $$\Delta I(t)=2\sqrt{I_{1}I_{2}}\cos(\Delta \varphi (t)+\varphi_{0})$$

where $I_{1}$ is the intensity of light entering the coupler via the sensing fiber. $I_{2}$ is the intensity of light entering the coupler via the reference fiber. $\varphi_{0}$ is the constant interferometric phase, determined by steady-state or slowly varying metastatic parameters such as the unbalanced length of the two interferometer arms, temperature, steady-state stress in the interferometer arms and steady-state stress in the laser; $\Delta \varphi (t)=\Delta \varphi_{1}(t)+\Delta \varphi_{2}(t)$ is the constant phase of the interferometric light caused by vibration acting on the DFB-FL or the interferometer arms.

When vibration acts on the grating region, the relationship between the output laser wavelength and axial stress can be calculated by the following equation:

(2) $$\Delta \lambda (t)=\frac{\lambda \sigma_{1}(t)}{E}\left\{1-\frac{n^{2}}{2}\;[P_{12}(1-\nu )-{P_{1}}_{1}\;\nu ]\right\}$$

When considering radial pressure, the wavelength variation formula can be derived as:

(3) $$\Delta \lambda (t)=\lambda \frac{P(t)}{Y}(a-b-c)$$

In Equation (3), the values of parameters *a*, *b*, and *c* are defined as follows:

(4) $$\left\{\begin{array}{c} a=2\nu \\ b=n^{2}p_{12}\nu \\ c=\frac{n^{2}}{2}\left(1+\nu \right)\left(1-2\nu \right)\left(p_{11}+p_{12}\right) \end{array}\right.$$

In the above equation, λ is the steady-state output wavelength. Δλ, is the wavelength change, n is the relative refractive index of the gain medium, $\sigma_{1}$ is the axial stress on the laser, E is the Young modulus of the fiber (the Young modulus of silica fiber is typically $6.5\times 10^{10}\;N/m^{2}$), ν is the Poisson ratio of silica fiber (typically 0.17), and $P_{11}$ and $P_{12}$ are the corresponding coefficients in the elasto-optic coefficient matrix. Substituting typical parameter values into the above equation reveals that the sensitivity of the optical fiber to axial stress is much higher than that to radial stress.

After the information carried by the vibration demodulated by the interferometric structure, the phase fluctuation induced by grating region strain is given by:

(5) $$\Delta \varphi_{1}(t)=\frac{2\pi nL}{\lambda^{2}+\lambda \Delta \lambda (t)}\Delta \lambda (t)$$

where *L* is the optical path difference between the two interferometer arms.

When vibration acts on the interferometer arms, the phase change of the light entering the coupler can be calculated by the following equation [26,27,28]:

(6) $${\Delta \varphi }_{2}(t)=2\left(1-n^{2}\left[\left(1-\nu \right)p_{12}-\upsilon p_{11}\right]/2\right)n_{f}kL_{S}\frac{\sigma_{2}(t)}{E}$$

where k is the wavenumber, $L_{S}$ is the interaction length of the vibration signal, $n_{f}$ is the refractive index of silica fiber, and $\sigma_{2}$ is the axial stress on the interferometer arms.

## 3. Analysis of the DFB-FL/Interferometer Composite Partial Discharge Test System

### 3.1. Noise Analysis of the DFB-FL/Interferometer Composite Partial Discharge Test System

**Figure 3:**
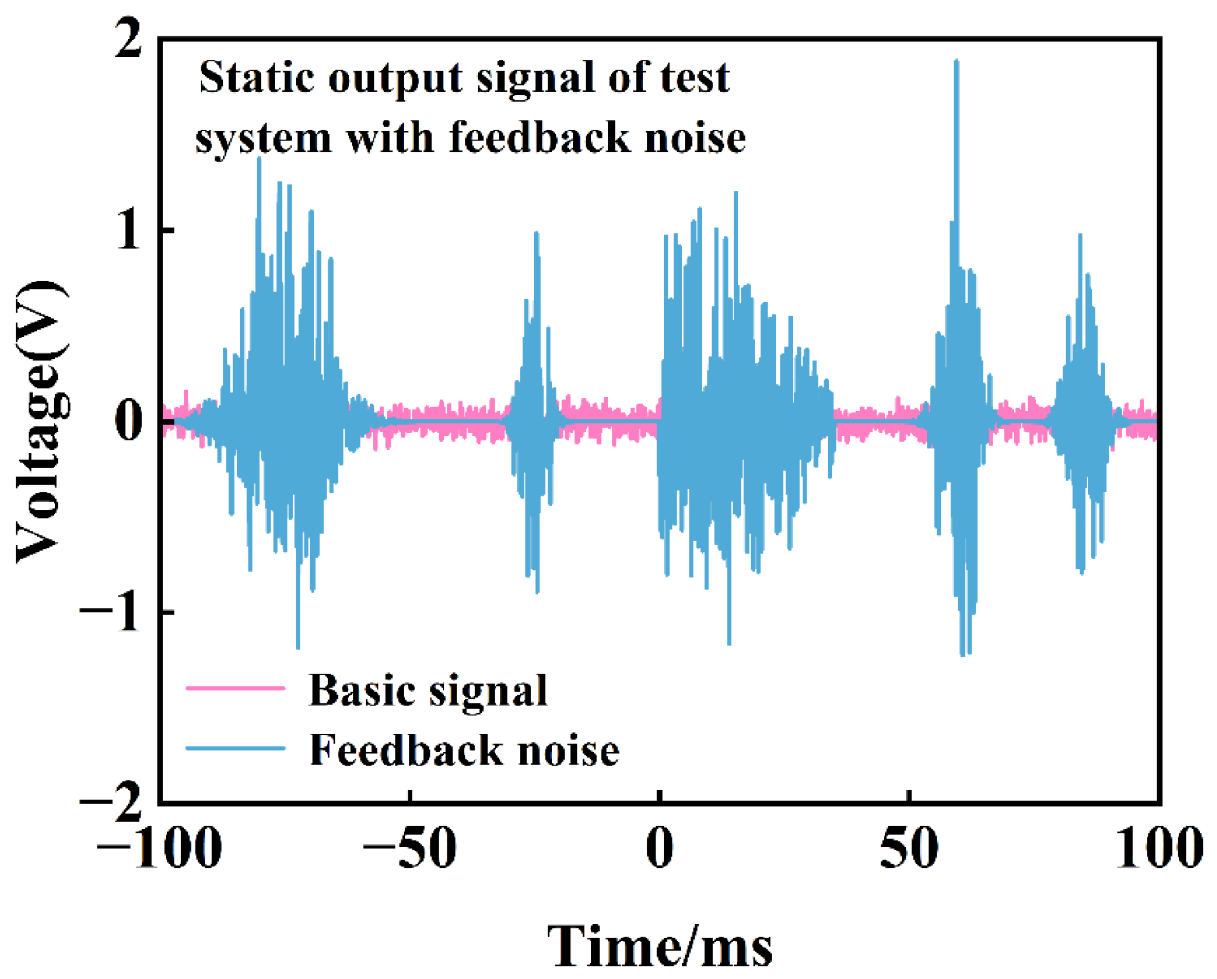
Typical feedback noise of the test system.

The internal noise of the test system mainly includes laser intensity noise and external optical feedback noise. The laser intensity noise primarily originates from the transfer of intensity noise from the 980 nm pump laser and the relaxation oscillation caused by periodic increases and decreases in the population inversion during the pumping process. This oscillation frequency is related to the pump rate, which is controlled by the pump power. Currently, the common doping methods for DFB-FLs emitting in the 1550 nm band are erbium-doped and erbium–ytterbium co-doped configurations. Compared with pure erbium-doped DFB-FLs, erbium–ytterbium co-doped DFB-FLs introduce multiple energy transfer mechanisms, including radiative energy transfer, non-radiative energy transfer, and phonon-assisted energy transfer between ytterbium and erbium ions, resulting in higher absorption efficiency for 980 nm pump light and more effective excitation of erbium ions. The intensity of the 980 nm pump laser responds to acoustic signals several times more strongly than the 1550 nm laser. The unabsorbed 980 nm laser from the right side of the DFB-FL enters the photodetector together with the 1550 nm laser and affects the measurement results. To achieve long-distance and high-sensitivity monitoring, the transmission fiber and interferometer arms are usually relatively long. Due to Raman scattering, 1550 nm laser feedback into the laser cavity is inevitably generated in the test system, and the accumulated feedback light readily produces the external optical feedback noise shown in Figure 3.

### 3.2. Improvement Scheme for the DFB-FL/Interferometer Composite Partial Discharge Test System

**Figure 4:**
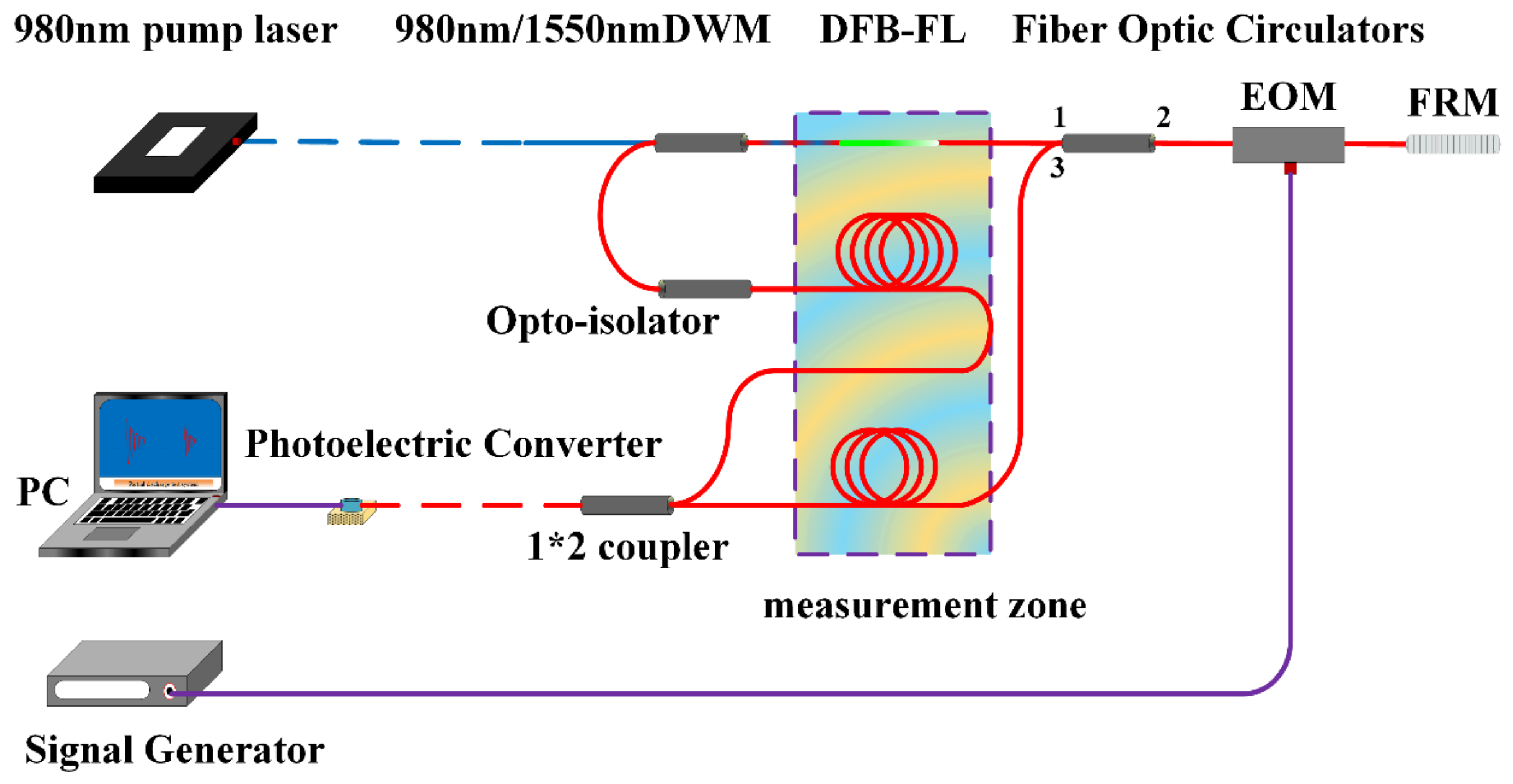
Feedback optical isolation combined with phase modulation method.

This paper proposes a system improvement scheme to address the above problems, as shown in Figure 4, which combines optical isolation technology with phase modulation. The output light from one side of the DFB-FL is introduced into port 1 of the fiber circulator. The laser exits from port 2 and successively enters an electro-optic phase modulator (EOM) or acousto-optic phase modulator (AOM) and a Faraday rotator mirror (1550 nm). After modulation and reflection, the light re-enters the circulator and exits from port 3 into the interferometer arms placed in the region under test. This structure uses a Faraday rotator mirror at a specific wavelength to filter out the residual 980 nm laser and it employs a fiber circulator with isolation capability together with an optical isolator to eliminate feedback light caused by Raman scattering in the long interferometer arms. It then adopts active phase tracking or the phase-generated carrier (PGC) method via the phase modulator to handle operating point drift. Both active phase tracking and PGC methods are already relatively mature theories and schemes in the field of interferometric demodulation. However, it should be noted that this scheme requires introducing a signal generator, a phase modulator, and metal wires for transmitting modulation signals outside the fiber optic system, which is clearly difficult to implement for long-distance, small-space application scenarios such as submarine cable joints. Therefore, the application of phase modulation technology to solve operating point drift still has its limitations in practical scenarios.

### 3.3. Grating Region Sensitivity Enhancement Characteristics of DFB-FL/Interferometer

**Figure 5:**
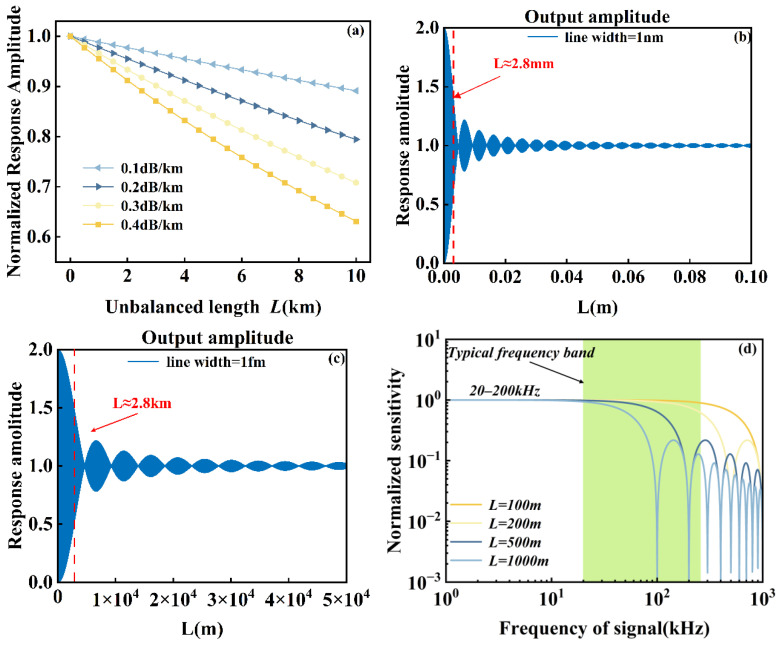
(**a**) Output power and coherence degree versus unbalanced length for DFB-FLs with different output linewidths. (**b**,**c**) Output power and coherence length vs. unbalanced length for DFB-FLs with different output linewidths. (**d**) Effect of acousto-optic coherence on test system sensitivity.

According to Equation (4), increasing the optical path difference between the two-interferometer arms to amplify the output signal amplitude per unit strain through the interferometer is the most straightforward sensitivity enhancement scheme for fiber grating strain sensors based on interferometric demodulation. By substituting typical parameters into the theoretical model for simulation, this paper obtains the curves of output light intensity and coherence as functions of unbalanced length under different linewidth conditions, as shown in Figure 5a. The curves are damped oscillatory, with only the envelope primarily presented in the figure. The analysis results indicate that as the unbalanced length increases, the amplitude envelope of the normalized output light intensity exhibits a monotonic decay characteristic, and the coherence gradually decreases as well. This phenomenon leads to a reduction in the signal-to-noise ratio and detection sensitivity of the actual measurement system. The decay rate of the response amplitude envelope is strongly correlated with the output linewidth of the laser: the narrower the linewidth, the slower the decay of the amplitude envelope; the results are as shown in Figure 5a–d. Clearly, when the grating region is regarded as the sensing structure and the interferometer arms serve as the demodulation structure, a longer unbalanced length of the interferometer arms can effectively improve the measurement sensitivity of the grating region. However, three factors limit the unlimited increase of the unbalanced length. The first is laser attenuation in long optical fibers. As shown in Figure 5a, an increase in the unbalanced length causes differences in the laser intensity entering the coupler, thereby reducing the output interferometric light intensity. Under a given system noise level, this reduces the signal-to-noise ratio of the measurement. The second factor is the laser linewidth. Whether the interferometer serves as a demodulation structure or a sensing structure, its theoretical sensitivity limit is controlled by the laser output linewidth. Figure 5b,c show the output light intensity and coherence degree under different linewidth conditions. The output light intensity curves are densely packed decaying oscillation curves, with the envelopes mainly shown in the figures. The interferometric signal amplitude decreases to half of that when the interferometer arms and reference arms are of equal length. When the laser output linewidth decreases to 1 fm, the unbalanced length reaches 2.8 km and the interferometric signal amplitude decreases by 3 dB. Taking the 3 dB amplitude reduction limit and the DFB-FL with a typical output linewidth of 35 kHz (approximately 0.395 fm) as an example, the unbalanced length can be extended to 7.1 km, which provides considerable convenience for engineering practice. The third factor limiting unlimited extension of the unbalanced length is the response intensity to signals of different frequencies. By substituting typical parameters into the coherence sensitivity formula $\Gamma =sinc[\frac{n_{eff}\pi f\left(L_{l}-L_{r}\right)}{c}]$ for simulation, as shown in Figure 5d, an excessively long unbalanced length reduces the sensitivity for vibration signal measurement in the high-frequency band. For the common frequency band of polymer insulation partial discharge, the ideal unbalanced length is below 200 m.

As mentioned above, the grating is far more sensitive to axial strain than to radial stress. Therefore, using a coupling medium to convert the strain from the object under test into axial strain acting on the optical fiber as much as possible is one of the effective sensitivity enhancement schemes. According to Equation (2), larger strain corresponds to greater output wavelength change, and a sufficiently large wavelength change can compensate for the deficiency in sensitivity at the optical static operating point.

### 3.4. Enhancement of Interferometer Arm Sensitivity for DFB-FL/Interferometer

**Figure 6:**
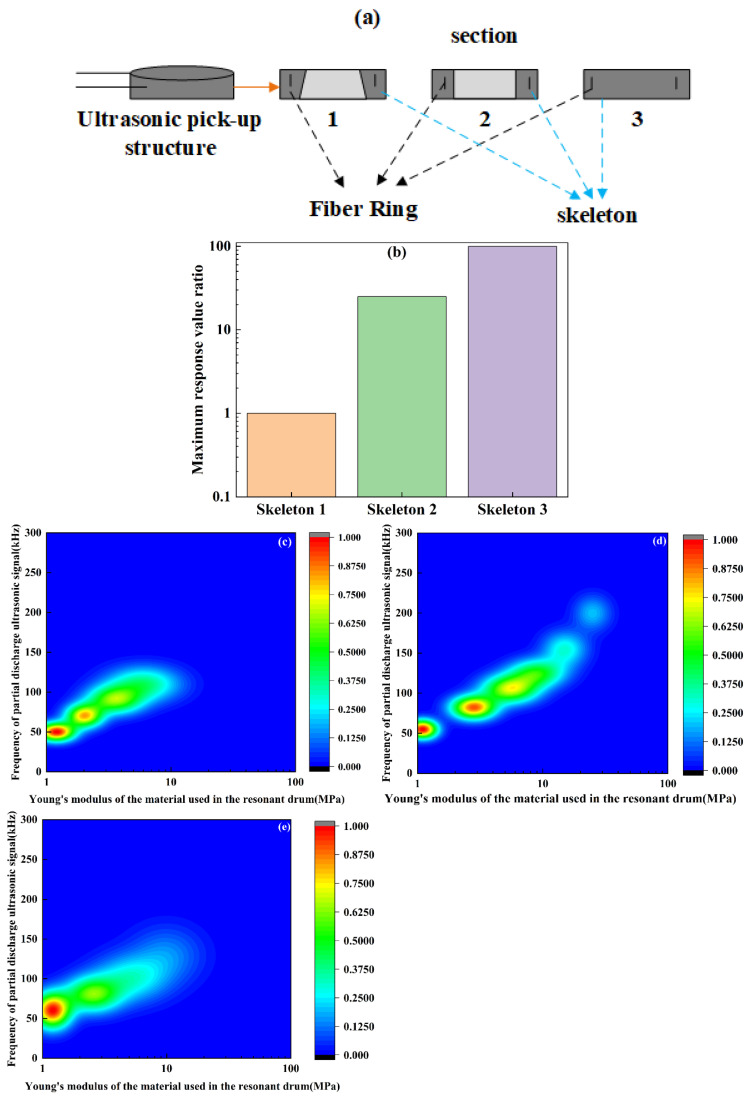
(**a**) Skeleton of three fiber-embedded sensing structures. (**b**) Maximum response ratio of three types of skeletons. (**c**–**e**) Contour maps of signal frequency varying with Young’s modulus.

Increasing the interaction length between acoustic signals and the sensing fiber by winding fiber loops is one of the main methods used to improve the measurement sensitivity of the interferometer. Increasing the number of fiber loop turns generally enhances the measurement sensitivity and broadens the sensing bandwidth. However, in solid-insulation partial discharge monitoring, vibration signals cannot effectively transmit vibration waves to every turn of the fiber as in liquid insulation. In this case, the skeleton connecting the object under test to the fiber loop becomes very important. This paper designs three fiber-embedded sensing structures as shown in Figure 6a. Using finite element simulation with the Young modulus of the skeleton material as a variable, the response sensitivity of the sensing structures to acoustic signals is calculated. Figure 6b shows the maximum response amplitudes of the three structures under the same excitation, with structure 1 as the reference. The simulation results are shown in Figure 6c–e, which are contour maps of the normalized sensitivity of the three structures varying with the Young modulus of the skeleton material and the signal frequency. From Figure 6, the following conclusions can be drawn. First, the solid skeleton exhibits higher response values. Second, although skeletons of different materials have different resonant frequencies, low-modulus skeletons are generally favorable for vibration signal detection. Third, all three structures tend to detect low-frequency signals, with their optimal response frequency bands around 50 kHz. The skeleton structure parameters are listed in Table 1.

## 4. DFB-FL/Interferometer Composite Partial Discharge Test System Response and Discharge Test

### 4.1. Experimental Platform for Partial Discharge Detection of Submarine Cable Intermediate Joints

**Figure 7:**
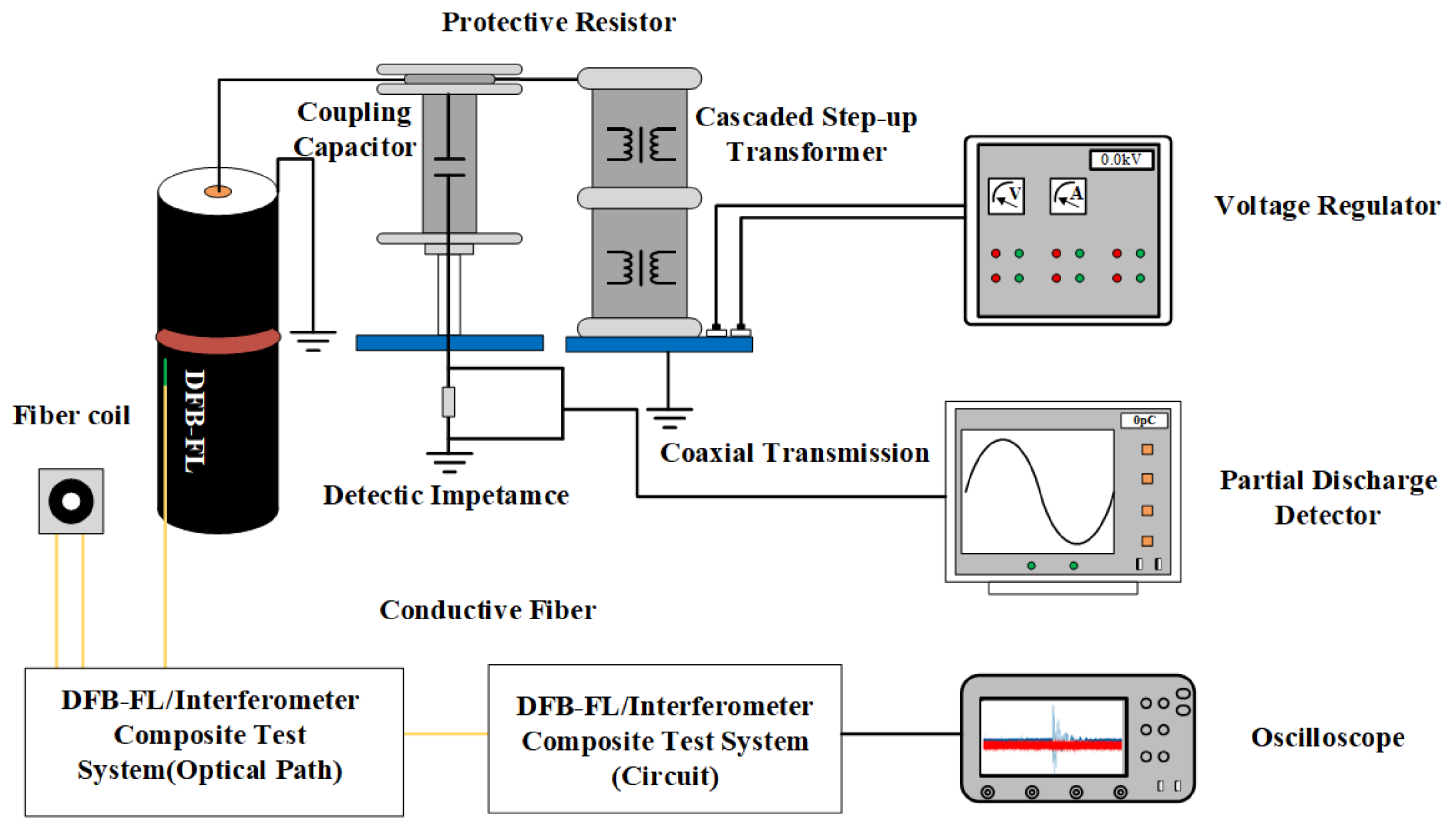
Cable joint partial discharge monitoring experimental platform.

Figure 7 shows the experimental platform developed by this research institute. It presents the test site, defective joint, sensor arrangement, and cross-sectional view of the defect after testing. To investigate the effect of sensing unit placement on test performance, two sets of experiments were carried out sequentially in this study. For the first set, the sensing unit was mounted on the outer semi-conductive sheath of the submarine cable after the semi-conductive layer of the joint was restored. Upon completion of the first set, the test system was disassembled, a lead sheath was fabricated, and the sensing unit was then placed on the outer surface of the lead sheath for the second set of experiments.

To separately verify the partial discharge detection performance of the grating region and fiber coil within the distributed feedback fiber laser (DFB-FL)/interferometer hybrid test system, each experimental set was divided into two independent tests. During the experiments, the pulse current method and the proposed DFB-FL/interferometer hybrid test system were applied for synchronous comparative monitoring. After testing, the cable joint was sectioned near the discharge location identified by the DFB-FL test. Following immersion in silicone oil, internal bubble inspection was performed to confirm whether the discharge originated from internal bubble defects.

### 4.2. Experimental Results of Partial Discharge on Submarine Cable Intermediate Joints

**Figure 8:**
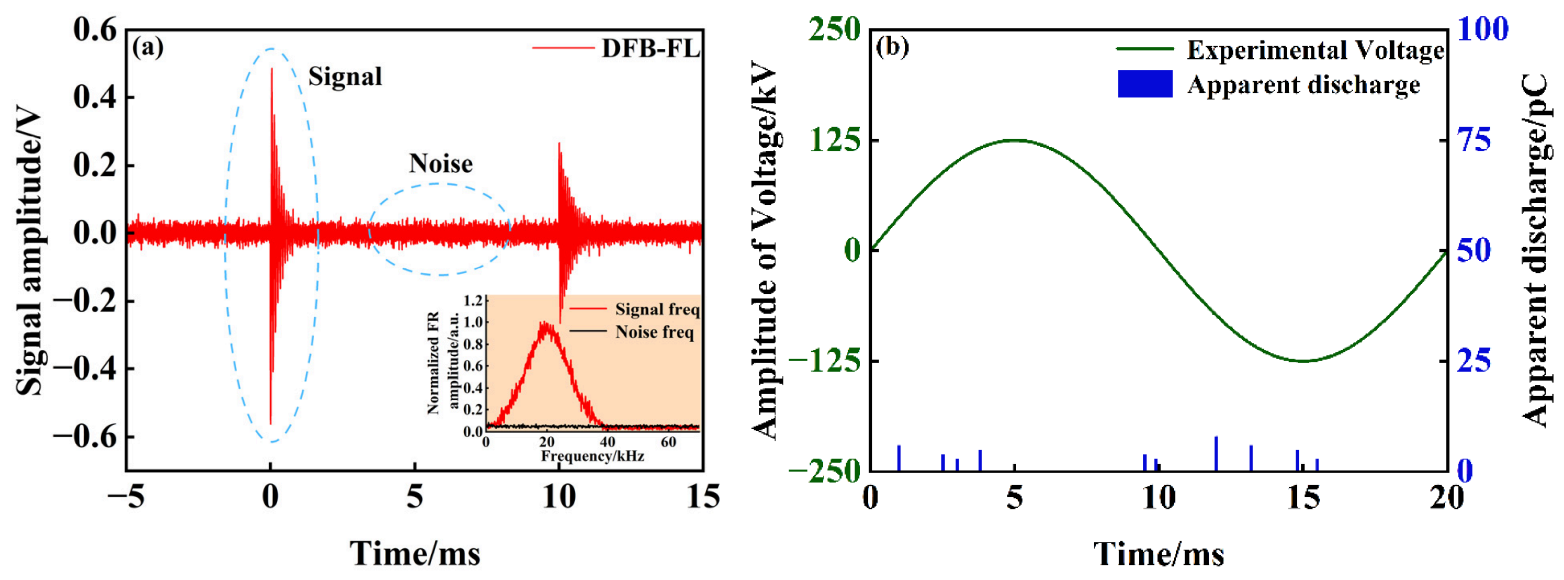
Response signal of the test system (DFB-FL) and 5 s cumulative discharge phase diagram at 90.5 kV test voltage. (**a**) Test system (DFB-FL) signal. (**b**) Five-second accumulated discharge phase spectrum.

Figure 8a shows the time-domain signal within one power frequency cycle measured by the testing system (grid region) under an experimental voltage of 90.5 kV, and Figure 8b shows the cumulative discharge phase-resolved pattern over 5 s under the same voltage. The test results indicate that within one cycle at 90.5 kV, there is an approximately 10 ms time interval in the signal concentration region measured by the DFB-FL/interferometer hybrid testing system in the grid region, which can reflect the discharge phase characteristics of bubble discharges to some extent. The signal frequency is mainly concentrated around 20 kHz (the inset shows the spectrum after Fast Fourier Transform). Since the response spectrum range of the grid region far exceeds the signal frequency obtained at this time, the signal fidelity is high. This proves experimentally that the partial discharge acoustic signal is indeed dominated by low-frequency acoustic signals and the minimum measurable discharge dropped to 6.75 pC for the grating region.

Along with the cumulative discharge phase-resolved pattern over 5 s at this voltage. Compared with the grid region, the fiber optic loop on the interferometer arm exhibits longer oscillation intervals and lower signal frequencies. At this point, the frequency of the oscillation signal is mainly determined by the natural frequency of the fiber optic loop and the minimum measurable discharge dropped to 12.66 pC for the fiber coil.

**Figure 9:**
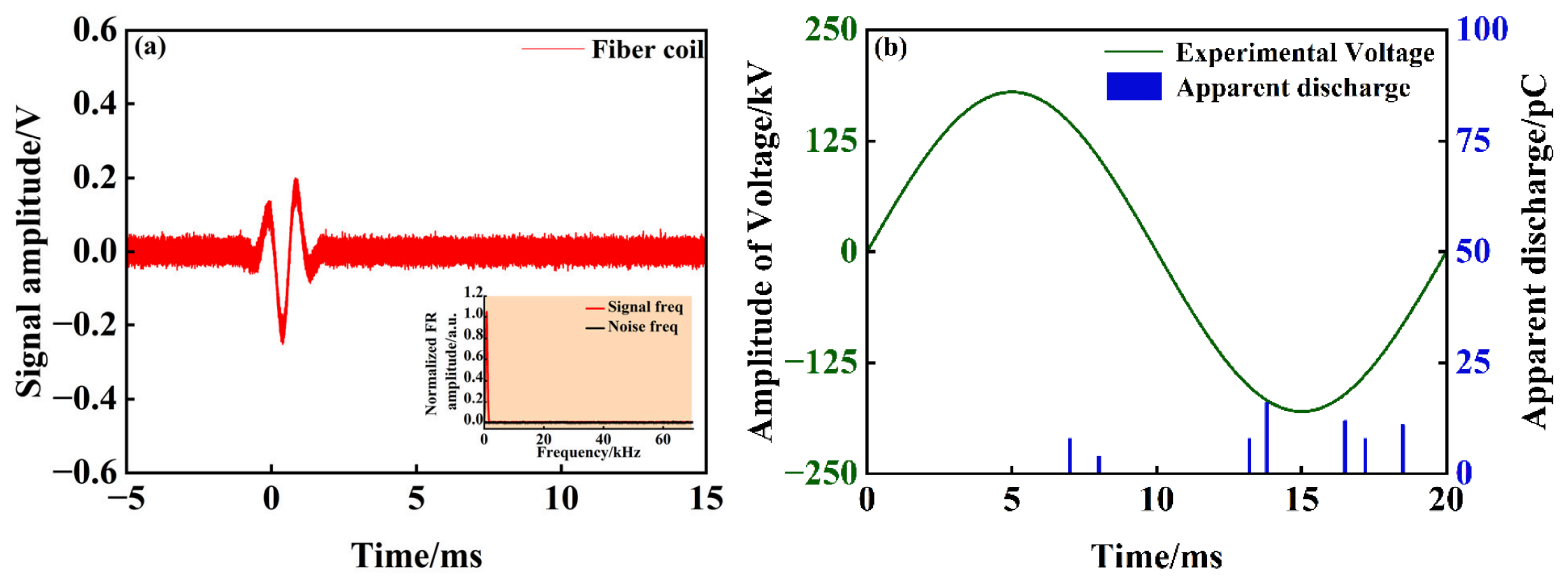
Response signal of the optical fiber loop and 5 s cumulative discharge phase diagram at 132 kV experimental voltage. (**a**) Test system (fiber ring) signal. (**b**) Five-second accumulated discharge phase spectrum.

Figure 9 shows the response signal of the optical fiber loop and the corresponding cumulative discharge phase-resolved pattern at 132 kV experimental voltage. Compared with the grid region, the fiber optic loop on the interferometer arm exhibits longer oscillation intervals and lower signal frequencies. From the test results, the grating zone exhibits both a lower inception discharge voltage and a lower corresponding discharge magnitude than the interferometer arm, indicating significantly better performance. However, the interferometer arm was originally designed to extend the testing range. That addressed the core limitation of point-type measurements—namely, their limited coverage in semi-crystalline polymers. Therefore, despite its slightly lower sensitivity, it remains of great significance for partial discharge detection in intermediate joints of long-length high-voltage submarine cables.

**Figure 10:**
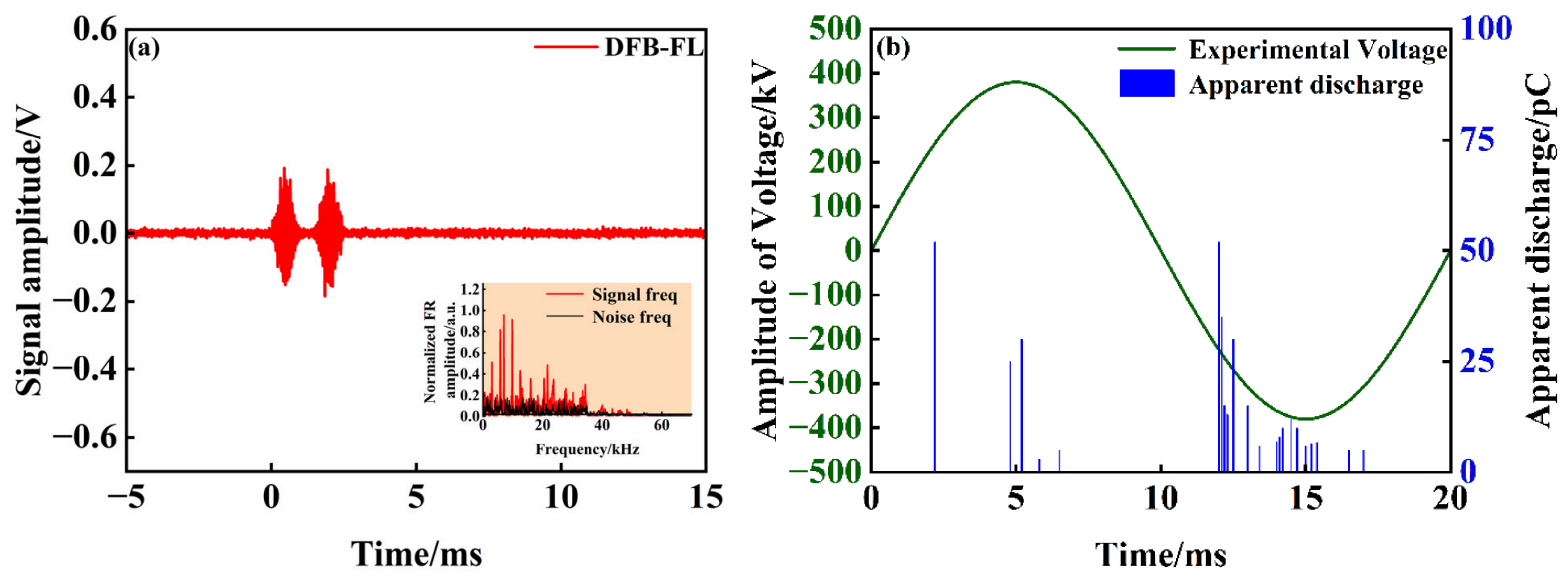
Signals from the testing system (DFB-FL) for needle puncture defects at 274 kV experimental voltage. (**a**) Test system (DFB-FL) signal. (**b**) Five-second accumulated discharge phase spectrum.

The time-domain waveform of the testing system’s grid region at the inception discharge voltage, along with the corresponding discharge phase-resolved pattern at that voltage, is shown in Figure 10a,b. From Figure 10b, it can be seen that when the sensor is placed outside the lead sheath, its spectral response characteristics are similar to those when it is placed outside the outer semi-conductive layer; both exhibit low-frequency signals below 60 kHz and the minimum measurable discharge quantity was 53.2 pC for the grating region.

**Figure 11:**
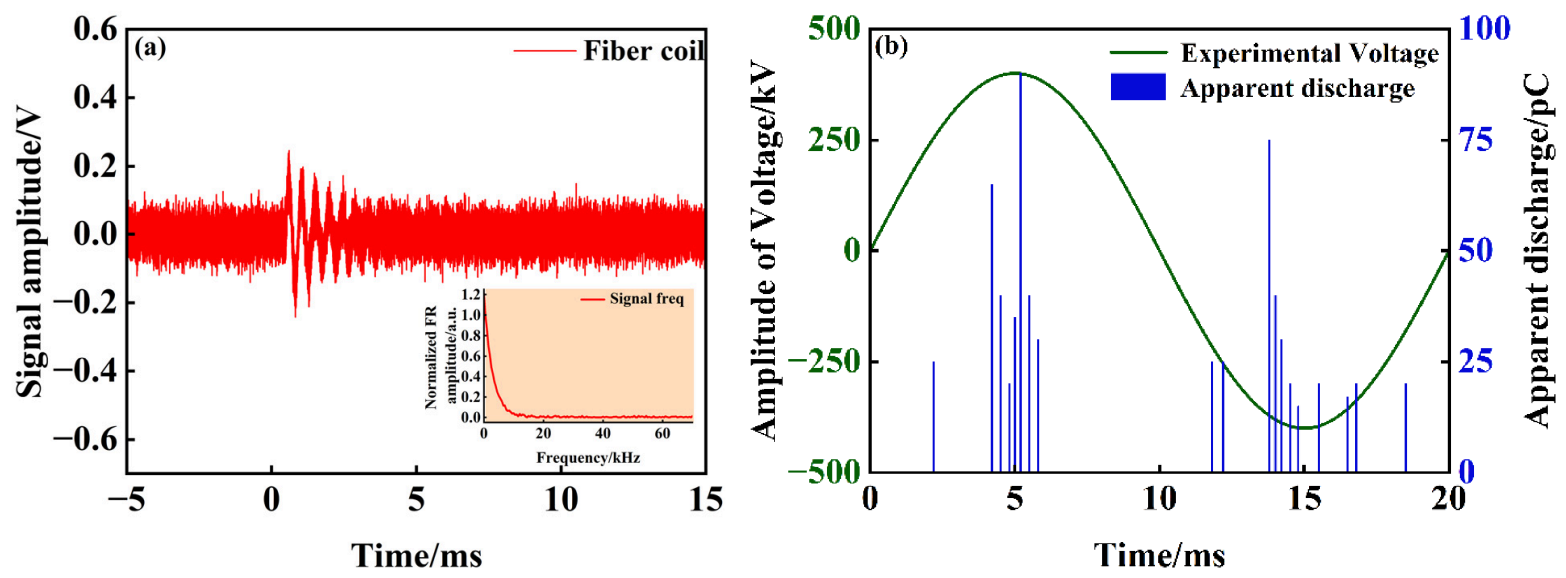
Response signal of the optical fiber loop and 5 s cumulative discharge at 274 kV. (**a**) Test system (fiber ring) signal. (**b**) Five-second accumulated discharge phase spectrum.

Figure 11a,b show the time-domain waveform of the testing system’s interferometer arm at the inception discharge voltage during the experiment, along with the corresponding discharge phase-resolved pattern at that voltage. Figure 11a indicates that the fiber optic loop of the testing system, due to the long duration and low frequency of the signals it perceives, is unable to effectively observe the discharge frequency. From Figure 11b, it can be seen that the occurrence of discharge phenomena is already very dense at this point. The minimum measurable discharge quantity was 89.6 pC for the fiber coil. However, as mentioned earlier, the original design intention of the testing system was to extend the measurement range, address the core limitation of point-type measurements—namely their limited coverage in semi-crystalline polymers—and find a viable partial discharge monitoring scheme for the demanding test environment of high-voltage submarine cables. Therefore, although the interferometer arm still cannot accurately count the discharge frequency like electrical measurement methods, it remains an effective partial discharge-monitoring scheme for the specific application scenario of high-voltage submarine cable.

## 5. Conclusions

This paper proposes a remotely demodulated quasi-distributed partial discharge (PD) detection system based on an all-fiber interferometer/DFB-FL composite PD testing system. The main conclusions are as follows:

1. The operating principle and noise influence mechanism of the remote sensing and interferometric demodulation system are analyzed. Theoretical simulations are performed to investigate the variations in output optical intensity and coherence degree with different unbalanced arm lengths, and the effects of laser attenuation and linewidth on coherence degree are also examined. For a DFB-FL (distributed feedback fiber laser) linewidth of 35 kHz, the maximum incoherent length can reach 7.1 km; however, for the PD frequency band, the optimal unbalanced length is below 200 m.
2. By employing the DFB-FL grating region and the interferometer arms, the system achieves multi-point distributed measurement, effectively addressing the issue of insufficient test coverage for semi-crystalline polymeric insulation materials, such as those used in high-voltage submarine cable factory joints. To enhance the measurement sensitivity of the interferometer, the interferometer arms are designed as fiber coil structures. Through simulation and experimental studies on three types of coil configurations, it is demonstrated that the solid-wound fiber coil achieves the highest response amplitude and the widest frequency response range; among the three structures, the solid-wound coil also exhibits the most prominent maximum response ratio.
3. In PD tests on a 220 kV high-voltage submarine cable factory-joint specimen, when the sensor unit is installed inside the metallic cable sheath, the minimum measurable discharge level is 6.75 pC for the grating region and 12.66 pC for the fiber coil. When the testing system is installed outside the metallic sheath, the minimum measurable discharge levels are 53.2 pC for the grating region and 89.6 pC for the fiber coil. Further research on this technology should be pursued to explore new technical pathways for online PD monitoring of high-voltage submarine cable factory joints.

## Figures and Tables

**Table 1:** Parameters of the three fiber-embedded sensing structures.

	Frame 1	Frame 2	Frame 3
Frame Height	5 mm	5 mm	5 mm
Outer Diameter	40 mm	40 mm	40 mm
Inner Diameter	10–36 mm	36 mm	0 mm

## Data Availability

The original contributions presented in this study are included in the article. Further inquiries can be directed to the corresponding author.
